# A37 OPTIMIZING ENDOSCOPIC SCHEDULING USING THE SMSA SCORE FOR ENDOSCOPIC MUCOSAL RESECTION.

**DOI:** 10.1093/jcag/gwae059.037

**Published:** 2025-02-10

**Authors:** D Maillet, N Chapelle, M Martel, C Menard

**Affiliations:** Universite de Sherbrooke, Sherbrooke, QC, Canada; Universite de Sherbrooke, Sherbrooke, QC, Canada; Research Institute of the McGill University Health Centre, Montreal, QC, Canada; Universite de Sherbrooke, Sherbrooke, QC, Canada

## Abstract

**Background:**

Colonoscopies prevent colorectal cancer (CRC) by detecting and removing neoplastic polyps. Complex polyps (CP) pose many challenges. While endoscopic mucosal resection (EMR) is the standard treatment, its procedure time is unpredictable, leading to scheduling inefficiencies. The SMSA (Size, Morphology, Site, Access) score helps classify CP and predict clinical risks but has never been used as a predictor of procedural time. A tool to predict EMR duration is needed to improve efficiency in resource-limited settings.

**Aims:**

We aim to assess if the SMSA and SMSA+ scores can predict procedural time and to determine whether the specific domains of the score, as well as additional pre-procedure factors, are associated with increased procedural duration.

**Methods:**

We conducted a retrospective cohort study at a tertiary-care center in Sherbrooke, Canada, involving consecutive adult patients who underwent EMR for CP between November 2020 and August 2024. The primary outcome is to evaluate if the SMSA and SMSA+ scores can estimate procedure duration. Secondary outcomes include identifying variables associated with procedure time. Data on demographics, polyp characteristics, procedure details, and adverse events were collected and analyzed using descriptive statistics and regression models.

**Results:**

A total of 100 patients were included (mean age: 67.9 ± 9.3, 53% female, Charlson score: 3.62 ± 1.98), The distribution of SMSA scores was as follows: Score 1: 0%; Score 2: 10.0% (mean time: 48.1 ± 15.3 mins); Score 3: 37.0% (mean time: 54.1 ± 15.7), and Score 4: 53.0% (mean time: 74.2 ± 30.5). The SMSA+ was distributed as score 0: 16.3% (mean time: 56.3 ± 16.4) and score 1: 83.7% (mean time: 66.3 ± 28.6). A mean of 2.3 ± 2.3 additional cold snare polypectomies were performed during the procedure with a similar distribution across all scores. Regression model indicated that both the SMSA score (P=13.3, t=3.0, p<0.01) and intra-procedural complications (P=29.9, t=3.9, p<0.01) were significant predictors of procedure time. Age, sex, Charlson score, fibrosis, fragmented specimen, and EMR technique did not significantly contribute to procedural time. Further analysis revealed that only the size domain of the SMSA score was associated with procedural duration (P=4.45, t=3.3, p<0.01). The SMSA+ score was not predictive of procedural time.

**Conclusions:**

The SMSA score, especially the size domain, is a useful predictor of EMR procedural time, while the SMSA+ score does not predict duration. These findings suggest that incorporating the SMSA score into clinical practice could enhance scheduling efficiency. However, further studies using multicenter data is needed to explore additional factors.

**Funding Agencies:**

None

